# The Maternal Disintegrative Responses Scale (MDRS): Development and initial validation

**DOI:** 10.1002/jclp.23414

**Published:** 2022-07-09

**Authors:** Miriam Chasson, Orit Taubman – Ben‐Ari

**Affiliations:** ^1^ School of Social Work Bar‐Ilan University Ramat Gan Israel

**Keywords:** dissociation, intrusive thoughts, mothers, postpartum, validation

## Abstract

**Objective:**

This study aimed to design and examine the validity of the Maternal Disintegrative Responses Scale (MDRS) to assess intrusive thoughts and dissociative experiences in the postpartum period.

**Method:**

A convenience sample of 455 mothers whose babies were up to 12 months old completed the MDRS and a series of questionnaires assessing postnatal depression (Edinburgh Postnatal Depression Scale [EPDS]), childbirth‐related post‐traumatic stress disorder (PTSD), obsessive‐compulsive disorder (OCD), and general symptoms of dissociation.

**Results:**

The final scale consists of eight items tapping two dimensions, intrusive thoughts and dissociative experiences, and displays good psychometric properties. Both factors were found to be related to EPDS, PTSD OCD, and general symptoms of dissociation. Primiparous women scored higher than multiparous women on both dimensions, and mothers of infants up to 3 months old scored higher on dissociative experiences than those whose infants were aged 4–12 months.

**Conclusions:**

The MDRS can contribute to the theoretical and practical conceptualization and assessment of these phenomena.

## 1 INTRODUCTION

During the postpartum period, the mother is faced with numerous changes at once, including physical, hormonal, familial, and psychological (Agrati & Lonstein, 2016). At the same time, she is required to invest considerable physical and mental resources in caring for an infant who is completely dependent on her. Along with the pleasure and satisfaction that usually accompany this experience, the high demands of early maternal caregiving and deep sense of responsibility for the vulnerable and helpless infant can also trigger stressful and negative experiences (Fonseca et al., 2018; Huth‐Bocks et al., 2016). In addition to depression and anxiety, which have been widely studied (Goodman et al., 2016; Silverman et al., 2017), a woman's negative experiences may be reflected in a wide range of disoriented and disintegrative responses, such as intrusive thoughts and dissociative experiences (Fairbrother & Woody, 2008; Lyons‐Ruth, 2003).

Intrusive thoughts, that is, thoughts and images with unwanted, unacceptable, or undesirable content, may be a prominent symptom of obsessive‐compulsive disorder (OCD; American Psychiatric Association, 2013). However, they are also very common in the general population, with the difference between normal and psychopathological intrusive thoughts being in their frequency, duration, intensity, and consequent level of distress. In addition, intrusive thoughts that characterize OCD are often accompanied by avoidant and compulsive behaviors aimed at dealing with the distress they evoke, while normal intrusive thoughts are experienced as unpleasant but tolerable (Rachman & De Silva, 1978).

In the postpartum period, intrusive thoughts generally take the form of unwanted aggressive reflections and images relating to harm to the infant caused by an external source, such as an accident, or by the mother herself (Brok et al., 2017; Leckman et al., 1999). Factors found to be associated with the appearance of intrusive thoughts at this time include a high level of stress, a low level of social support (Fairbrother & Woody, 2008), and prolonged infant crying (Fairbrother et al., 2015).

As noted above, intrusive thoughts at low frequency are common among women in the general population in the postpartum period, especially after the first birth, and do not usually constitute a clinical warning sign of any pathological disturbance in the mother or any actual injury she may cause (Fairbrother et al., 2019). However, an increase in their frequency, duration, intensity, and consequent distress and behaviors may indicate the development of OCD (Buchholz et al., 2020; Fairbrother et al., 2018).

Yet, despite the distress that may be aroused by maternal intrusive thoughts at varying levels of frequency and intensity, they have received little attention in the empirical, therapeutic, or social discourse (Brok et al., 2017). Ignoring this issue may impact the low detection of women with OCD, as well as the lack of awareness and the taboo surrounding the normal occurrence of intrusive thoughts, potentially leading to feelings of shame and guilt among those who experience them (Boyd & Gannon, 2021; Meehan et al., 2021).

**Figure 1:**
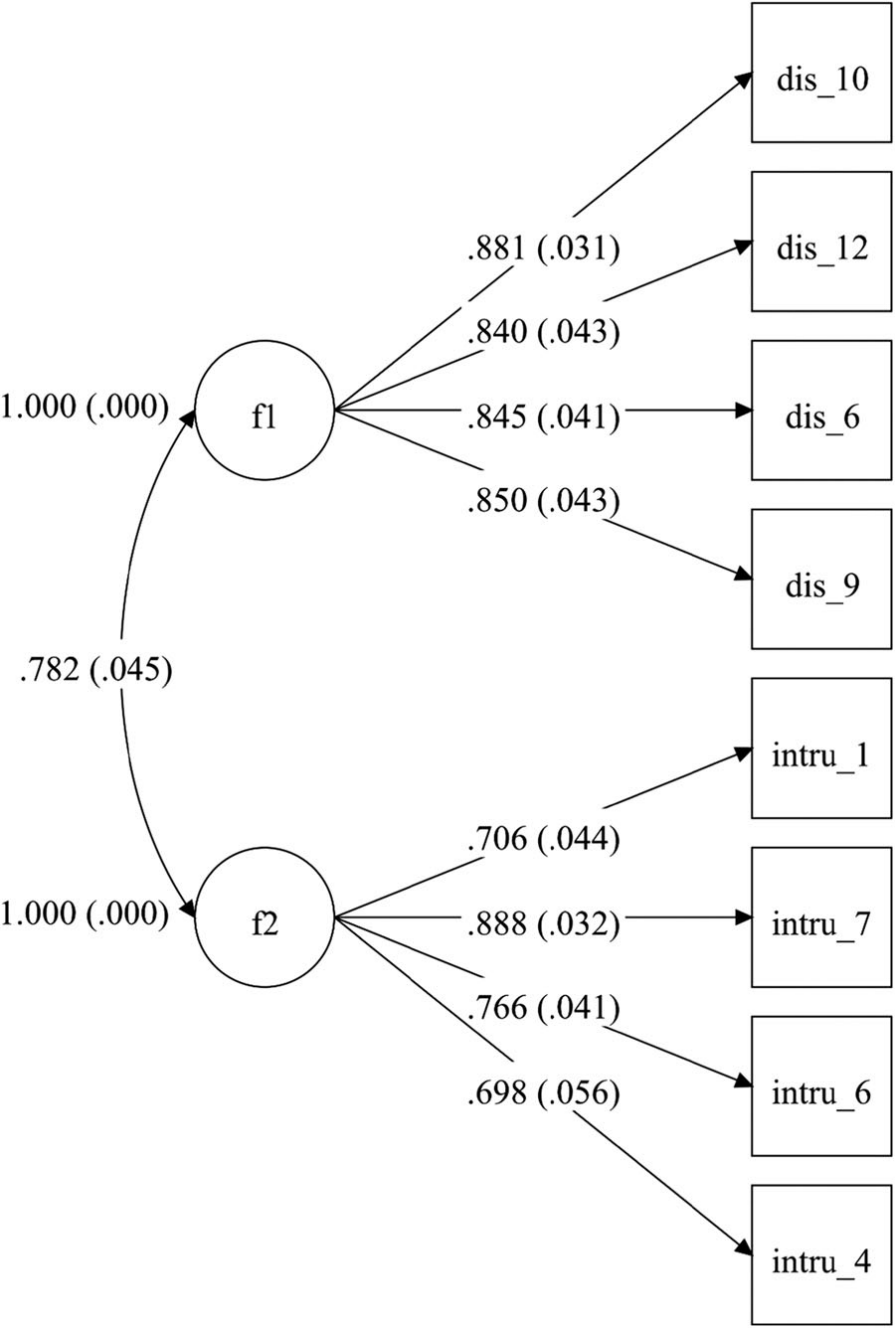
Confirmatory factor analysis (CFA) standardized coefficients (Sample 2)

There are few self‐report tools for examining OCD among women after childbirth that also tap intrusive thoughts. The two prominent existing questionnaires are the Perinatal Obsessive‐Compulsive Scale (POCS; Lord et al., 2011), and the Parental Thoughts and Behaviors Checklist (PTBC; Thiséus et al., 2019). The items in these questionnaires contain detailed descriptions of thoughts about injury to the infant caused by an external factor or by the mother herself, as well as repeatedly checking up on the infant and avoidant behaviors related to intrusive thoughts. Thus, while these tools are important for assessing intrusive thoughts that indicate OCD, they are less suited to examining intrusive thoughts in the normative range, and might be discounted entirely by women who do not see themselves in the descriptions.

In addition to these questionnaires, the Postpartum Distress Measure (PDM; Allison et al., 2011) contains four items relating to the repetitiveness of thoughts and behaviors among postpartum women, as expressed in OCD. We therefore believed there was a need to develop a new questionnaire that would tap a broader range of intrusive thoughts in general, not necessarily as a sign of OCD, but as a normative disintegrative response of a mother contending with infant care.

Another expression of disintegrative responses among women in the postpartum period is dissociative experiences. According to the *Diagnostic and Statistical Manual of Mental Disorders fifth edition* (DSM‐V), dissociation may be reflected in a variety of situations and is characterized by disruption or discontinuity in the integration of consciousness, memory, identity, emotion, and perception of body representation (American Psychiatric Association, 2013). Dissociation is commonly considered a psychological defense mechanism applied in the face of stressful and overwhelming situations, such as traumatizing events, which allows a person to disconnect from painful emotions (Carlson, 1998; Liotti, 2006). However, similar to intrusive thoughts, dissociative experiences can occur across a broad spectrum from adaptive to pathological behavior. When such experiences occur at high frequency and intensity and disrupt normal functioning, they can be regarded as pathological. Yet dissociation may also be reflected in normative everyday experiences in the general population (Putnam, 1991).

Infant care in the postpartum period may trigger dissociative experiences in a mother for several reasons. The first is the helplessness she may feel in her maternal role (Huth‐Bocks et al., 2016; Solomon & George, 2011). Moreover, they may be the result of trauma, whether trauma related to the childbirth experience itself (Thiel & Dekel, 2020) or an unprocessed past trauma (Jacobvitz et al., 2006; Oh et al., 2016). Finally, the disrupted sleep that characterizes this period may also be related to greater dissociative experiences (Giesbrecht & Merckelbach, 2004).

Despite this evidence in the literature, however, studies examining dissociation in the context of motherhood, and specifically in the postpartum period are scarce. Furthermore, the few existing investigations have employed two main methods: self‐report questionnaires measuring general dissociative experiences (e.g., Lev‐Wiesel & Daphna‐Tekoah, 2010; Oh et al., 2016); and a coding system based on observations of mother–child interactions (Jacobvitz et al., 2006; Main & Hesse, 1990). However, to the best of our knowledge, no previous studies have used self‐report questionnaires specially designed to examine dissociative experiences in the postpartum period and their manifestation in a mother's daily experience of caring for her infant.

In addition to the lack of knowledge about specific disintegrative responses among mothers in the postpartum period, little is known about the potential relationship between them. The possibility of a connection between distinctive reactions to the maternal role is suggested by a series of studies conducted by Main and Hesse (1990, 1995), who identify three patterns of behaviors that may characterize mothers during their interactions with their infant: frightening behavior, expressed in sudden and frightening reactions to the child; frightened behavior, manifested in expressions of sudden fear by the mother; and dissociative behavior, reflected in the mother's physical or emotional stagnation and freeze reactions during the interaction. However, as these studies rely on the observation and interpretation of mothers' behavior by the researchers, they do not allow room for the women's own reports of their experiences.

In light of the lack of instruments relating specifically to maternal disintegrative responses during early maternal caregiving, we sought to develop a self‐report questionnaire that would examine a broad spectrum of mothers' intrusive thoughts and dissociative experiences as expressed in infant care in the postpartum period. Moreover, we wished to devise a friendly instrument that would encourage candid responses to what may be perceived as sensitive issues.

Thus, the aim of this study was to construct and validate a short bidimensional scale we named the Maternal Disintegrative Responses Scale (MDRS). In addition, given the heightened distress that may characterize the first experience of parenting and the parenting of an infant in their first months of life (Nelson et al., 2014; Reck et al., 2009), we examined differences on the two dimensions between primiparous and multiparous women, as well as between mothers whose infants were up to 3 months old and those whose infants were aged 4–12 months. Finally, for the purposes of examining convergent and discriminant validity, we examined the associations between the MDRS factors and psychopathological constructs, namely, postnatal depression, birth‐related post‐traumatic stress disorder (PTSD), OCD, and general symptoms of dissociation.

Due to their characterization as expressions of mothers' distress and negative feelings, we hypothesized that both intrusive thoughts and dissociative experiences would be related to postpartum depression and birth‐related PTSD. Furthermore, we predicted that intrusive thoughts would be more strongly related to OCD than dissociative experiences, and that dissociative experiences would be more strongly related to general symptoms of dissociation than intrusive thoughts.

## 2 METHOD

### 2.1 Item generation

Items for the MDRS were formulated on the basis of statements drawn from a preliminary qualitative study conducted by the researchers that dealt with mothers' early caregiving experiences (Chasson & Taubman – Ben‐Ari, 2020), as well as on existing tools relating to dissociative symptoms and intrusive thoughts in general (Trauma Symptom Inventory; Briere et al., 1995; Dissociative Experiences Scale; Bernstein & Putnam, 1986). This process resulted in a 20‐item scale, which, based on the literature relating to mothers' disintegrative responses (Jacobvitz et al., 2006; Main & Hesse, 1990), tapped two domains: maternal dissociative experiences (10 items); and maternal intrusive thoughts (10 items). We then asked a panel of four independent judges with experience in clinical and research work with women in the perinatal period to evaluate the clarity and coherence of the items. In view of the judges' recommendations, redundant or unclearly worded items were removed, leaving us with an initial pool of 14 items, 7 items per dimension.

### 2.2 Procedure

Following approval of the study protocol by the university's Institutional Review Board, convenience sampling was used to recruit participants. A request was posted on social media forums for mothers, asking women who had given birth in the past year to participate in the study. The electronic link to the questionnaire was provided. Participants were informed that all the details and data collected for the study would remain confidential and anonymous, and that they were free to terminate their participation at any stage, without any sanctions. Finally, they were told that in case of distress during or after completing the questionnaire, they could contact the researchers. Information regarding counseling services for women was attached as well.

### 2.3 Participants

Data were collected in two rounds, independently, using the same survey method (*n*1 = 248; *n*2 = 207). The total sample consisted of 455 women aged 21–47 years *(M* = 32.12, SD = 5.01) whose babies were up to 12 months old (*M* = 4.88, SD = 3.13). Ninety‐seven percent of the women were Jewish. The majority were married (92%), had a BA degree (61%), were on maternity leave at the time of the study (64.3%), defined their economic status as average (61%), and reported their health condition as very good (57.5%). In addition, 67% of the women had older children, with the number ranging from 1 to 6 (*M* = 2.18, SD = 1.22). Most of the women had given birth to a single infant (97.8%) and had undergone a vaginal birth (71.5%) at term (37 + weeks of pregnancy; 87%).

### 2.4 Measures

MDRS was used to assess the disintegrative responses of mothers of young infants. The scale consists of two dimensions: *intrusive thoughts* (seven items), that is, the mother's experience of unwanted and uncontrolled thoughts when with or caring for the baby (e.g., “When I'm holding the baby, the uncontrollable thought that I'm going to drop him/her flits through my mind”); and *dissociative experiences* (seven items), relating to the mother's feelings of detachment and alienation from herself, her baby, or reality when with or caring for the infant (e.g., “When I'm with the baby or caring for him/her, I feel as if I'm not really there but only watching from a distance”). Participants were asked to rate how often they had the experience described in each item, indicating their responses on a scale ranging from 0 (*never*) to 4 (*very often*).

The Edinburgh Postnatal Depression Scale (EPDS; Cox et al., 1987), a 10‐item questionnaire assessing depressive symptoms in women around the time of birth, was used to measure postpartum depression. Participants were asked to rate how often they had experienced the feeling described in each item in the past week (e.g., “I have been anxious or worried for no good reason”), marking their responses on a scale ranging from 0 (*never*) to 3 (*very often*). In this study, Cronbach's *ɑ* was 0.87. A score was computed for each participant by summing her responses to all items, with higher scores indicating higher depressive symptoms.

The PTSD Checklist for DSM‐5 (PCL‐5; Weathers et al., 2013), adapted for mothers after childbirth, was used to assess birth‐related posttrauma. The questionnaire examines the frequency of the 20 *DSM‐5* symptoms of PTSD following the childbirth experience. Participants were asked to indicate how often they had been bothered by the problem described in each item during the past month (e.g., dreams or nightmares about the birth), marking their responses on a scale ranging from 0 (*never*) to 4 (*very often*). In this study, Cronbach's *α* was 0.87. A score was computed for each participant by averaging her responses to all items, with higher scores indicating a higher level of posttraumatic symptoms following childbirth.

The Obsessive‐Compulsive Inventory (OCI‐R; Foa et al., 2002), an 18‐item questionnaire, was used to measure the presence and severity of OCD symptoms. Participants were asked to rate how distressed they had been by the experience described in each item during the past month (e.g., “I wash and clean obsessively”), indicating their responses on a scale ranging from 0 (*not at all*) to 4 (*extremely*). Cronbach's *α* in this study was 0.90. A score was calculated for each participant by averaging her responses to all items, with higher scores indicating a higher level of obsessive‐compulsive symptoms.

The Trauma Symptom Inventory; (Briere et al., 1995) was used to assess general dissociative symptoms. The questionnaire contains 100 items relating to global posttraumatic stress on 10 dimensions. In this study, only the dissociation subscale, consisting of 10 items (e.g., “Feeling that things are ‘unreal’”) was employed. Participants were asked to rate how often they had experienced the problem described in each item in the past month, indicating their responses on a scale ranging from 0 (*never*) to 3 (*often*). In this study, Cronbach's *α* for the 10 items was 0.92. A score was assigned to each participant equal to the mean of her responses to all items, with higher scores indicating greater frequency of dissociative symptoms.

A sociodemographic inventory was used to tap the characteristics of the mother and the infant (mother's age [continuous], parity [0 = primiparous; 1=multiparous], infant's age [continuous]).

### 2.5 Data analysis

To validate the expected bifurcation of the new scale, we applied exploratory factor analysis (EFA) in two forms: (1) continuous EFA and (2) categorical EFA. The Mplus V.8.3.1 ESEM procedure was used for both forms. The parallel analysis approach was used to determine the optimal dimensionality of the MDRS. Estimation of the parameters was based on the Weighted Least Square Mean and Variance (WLSMV) estimator, and oblique rotation was chosen to obtain more interpretable results by specifying a correlated structure given that the underlying hypothesized concepts were not expected to be completely independent. To allocate items to factors, a threshold of 0.40 for factor loadings was used as a guideline. Missing values were estimated from observed data, and replaced by means of multiple imputations, which employed the expectation maximization algorithm (EM). Model fit was tested on a second sample using the following indices: the *χ* ^2^ test for model fit (*χ* ^2^), *χ* ^2^/*df* ≤3 (Kline, 2016); the comparative fit index (CFI; Bentler, 1990; Hu & Bentler, 1999); the Tucker–Lewis Index (TLI; McDonald & Marsh, 1990); and the root mean square error of approximation (RMSEA; Browne & Cudeck, 1993). A good model fit was indicated by CFI and TLI ≥ 0.95, and RMSE*A* = < 0.08 (Brown, 2015; Hu & Bentler, 1999).

MacDonald's omega coefficients were then computed to test the internal consistency of the MDRS factors. To assess the validity of the new scale, Pearson's and Spearman's correlations between the MDRS factors and psychopathology variables were computed. This exploratory stage was complemented by a confirmatory stage and full structural equation modeling (SEM) to determine associations between the newly designed and adjusted scales and similar existing validated instruments.

### 2.6 Validation procedure

For the sake of simplicity, we used the two rounds for EFA and confirmatory (CFA) factor analysis. That is, Sample 1 was assigned to EFA and Sample 2 was then used for cross‐validation of the optimal CFA model, testing two alternative solutions.

## 3 RESULTS

### 3.1 Data screening

Analysis across the study variables revealed relatively low rates of missing values, ranging from as low as none to as high as 1.8%. Little's test for MCAR (missing completely at random; Little, 1988) indicated that values were MCAR, *χ* ^2^ (3239, *N* = 455) = 3015.026, sig.=0.998. A multiple imputation procedure using a stochastic regression model to impute random values for the missing values based on prior information about the data was then conducted. Five iterations were calculated to replace the mean score for continuous variables and the prevalent values for the categorical variables.

### 3.2 Establishing factor dimensionality with EFA—Sample 1

First, EFA was conducted on the initial 14‐item version of the MDRS. The results showed that the eigenvalue > 1.00 criterion suggested two factor dimensions (2nd dimension: eigenvalue = 2.00 > parallel eigenvalue = 1.31; 3rd dimension: eigen value 0.90 < parallel = 1.24) if items were considered continuous. Two factor dimensions were also identified by the categorical analysis based on model fit indices (CFI = 0.971, TLI = 0.959, RMSEA = 0.075, 95% confidence interval [CI]: [0.06,0.09]). Closer inspection of the factor loadings on the preferred two‐factor solution revealed that a reduced factor solution might exist. While still retaining the two dimensions, it contained a smaller number of items. This possibility was examined in two steps. First, to ensure that the two‐factor structure was the optimal solution, we extracted three dimensions, resulting in the exclusion of three items due to either multiple loadings or loadings smaller than 0.40 (Howard, 2016). Next, testing the two‐factor structure, another three items were dropped due to low loadings (two items) and multiple loading (one item). Thus, the final shorter form consisted of two dimensions of four items each, and evidenced above acceptable model fit (CFI = 0.999, TLI = 0.997, RMSEA = 0.028). A one‐factor solution based on the eight items was also tested to ensure that an alternative structure was inferior. The results of the analysis are presented in Table 1. For the purposes of the analysis, we considered the polytomous assumption by applying the WLSMV estimator (Muthén Bengt, 1983). Factor loadings are shown in Table 2. To test for internal consistency, we used a continuous approximation to assess the McDonald Omega (Hayes & Coutts, 2020), which yielded ω = 0.804 for dissociative experiences, and ω = 0.813 for intrusive thoughts. Pearson's and Spearman's correlation coefficients calculated between the dissociation and intrusion subscales (Pearson's *r* = 0.444; Spearman's *r* = 0.388) showed them to display a reasonable level of relationship while remaining distinct from one another.

**Table 1:** Model fit statistics for the single and two‐factor EFA models (Sample 1—*n* = 247)—8 items

Model	*χ* ^2^	*Df*	*χ* ^2^_/*df*_	CFI	TLI	RMSEA
Two factors	15.50	13	1.19	0.999	0.997	0.028
Single factor	190.37***	20	9.52	0.908	0.871	0.186

*Note*: *χ* ^2^ diff (*df* = 7) = 174.87, *p* < 0.001.

Abbreviations: CFI, comparative fit index; RMSEA, root mean square error of approximation; TLI, Tucker–Lewis Index.

*** *p* < 0.001.

**Table 2:** EFA factor loadings of the Maternal Disintegrative Responses Scale (MDRS)—(Sample 1—*n* = 247)

Item	Dissociative experiences	Intrusive thoughts
12. When I'm with the baby or caring for him/her, I feel as if I'm not really there but only watching from a distance.	**0.94** *	0.003
9. There are moments when I feel like a stranger to my own baby.	**0.88** *	−0.001
10. When I'm with the baby I feel that what's happening isn't real, as if it's a dream or a movie.	**0.90** *	−0.04
6. When I hold the baby, I feel as if it's not really me doing it.	**0.88** *	0.07
1. I think of things that could happen to my baby that aren't really logical.	−0.03	**0.88** *
7. I have unwanted thoughts about bad things that could happen to my baby.	0.27	**0.70** *
6. When I'm caring for the baby, disturbing thoughts suddenly pop into my head.	0.31*	**0.59** *
4. When I'm holding the baby, the uncontrollable thought that I'm going to drop him/her flits through my mind.	0.29*	**0.44** *

*Note*: Item scales were analyzed as categorical. Bold values indicate loading values for final factor items.

* *p* < 0.05.

### 3.3 Confirming construct validity with CFA—Sample 2

Complementary random sample data were employed to confirm the EFA results assuming polytomous rather than continuous item distribution. Goodness of fit was evaluated using the same indices described for the EFA analysis conducted on data from Sample 1. The following models were evaluated: (1) a single‐factor independent cluster model of CFA (ICM‐CFA), which is generally recommended to examine the hypothesis of maximum parsimony (Brown, 2015); and (2) a two‐factor ICM‐CFA model that emerged from the Sample 1 data. The fit indices of the two alternative CFA models are presented in Table 3. The single‐factor confirmatory model shows that the RMSEA value did not meet the required threshold (RMSEAR = 0.14 > 0.10), whereas CFI and TLI did. The two‐factor solution slightly improved on the single‐factor solution, that is, TLI and CFI were higher, RMSEA was below 0.10, and χ^2^/df was smaller than 3 (see Table 3 and Figure 1).

**Table 3:** Model fit statistics for CFA of a single and two‐factor MDRS models (Sample 2—*n* = 208)—8 items

	*χ* ^2^	*Df*	χ ^2^_/*df*_	CFI	TLI	RMSEA
Single factor	105.01***	20	5.25	0.948	0.927	0.143
Two factors (dissociative experiences and intrusive thoughts)	54.66***	19	2.88	0.978	0.968	0.095
Difference test	50.35***	1				

Abbreviations: CFA, Confirmatory factor analysis; CFI, comparative fit index; MDRS, Maternal Disintegrative Responses Scale; RMSEA, root mean square error of approximation; TLI, Tucker–Lewis Index.

*** *p* < 0.001.

### 3.4 Descriptive statistics

Table 4 presents the descriptive statistics of the MDRS subscales and the prevalence of each item (rates of non‐0). In total, 34.7% of the women reported having at least one dissociative experience, and 85.1% reported having at least one intrusive thought, in the past month. Inspection of the items' distribution parameters indicates that whereas the intrusive thoughts items are relatively normally distributed, those of the dissociative experiences are largely positively skewed, indicating relatively few positive answers on these items. Considering that the dissociative experiences described in these items are relatively extreme, these results are not surprising.

**Table 4:** Descriptive statistics of final MDRS subscales (*N* = 455)

Items	*M* (SD)	Skewness (Se)	Kurtosis (Se)	Range	Percent answering above 0 (*never*) %
12. When I'm with the baby or caring for him/her, I feel as if I'm not really there but only watching from a distance.	0.26 (0.67)	2.97 (0.11)	8.99 (0.23)	0–4	16.3
9. There are moments when I feel like a stranger to my own baby.	0.24 (0.66)	3.31 (0.11)	11.95 (0.23)	0–4	16
10. When I'm with the baby I feel that what's happening isn't real, as if it's a dream or a movie.	0.43 (0.82)	1.96 (0.11)	3.15 (0.23)	0–4	26.6
6. When I hold the baby, I feel as if it's not really me doing it.	0.27 (0.64)	2.62 (0.11)	6.88 (0.23)	0–4	18
1. I think of things that could happen to my baby that aren't really logical.	1.48 (1.20)	.29 (0.11)	−0.96 (0.23)	0–4	74
7. I have unwanted thoughts about bad things that could happen to my baby.	1.05 (1.07)	0.073 (0.11)	−0.46 (0.23)	0–4	60.2
6. When I'm caring for the baby, disturbing thoughts suddenly pop into my head.	1.23 (1.15)	0.47 (0.11)	−0.89 (0.23)	0–4	63.7
4. When I'm holding the baby, the uncontrollable thought that I'm going to drop him/her flits through my mind.	0.58 (0.89)	1.54 (0.11)	1.81 (0.23)	0–4	37.1
Total	0.69 (0.62)	1.25 (0.11)	2.18 (0.23)	0–4	85.3
Dissociative experiences	0.30 (0.57)	2.63 (0.11)	8.08 (0.23)	0–4	34.7
Intrusive thoughts	1.08 (0.85)	0.59 (0.11)	−0.39 (0.23)	0–4	85.1

Abbreviation: MDRS, Maternal Disintegrative Responses Scale.

### 3.5 Reliability and discriminant validity

The internal consistency of the 8‐item MDRS was evaluated using the total sample (*N* = 455). The McDonald's Omega coefficients for the dissociative experiences and intrusive thoughts subscales were 0.840 and 0.808, respectively, indicating acceptable reliabilities, which aligned with the internal consistencies calculated earlier. Table 5 shows the results of expanded SEM for the full sample (*N* = 455). This step was used to enhance the context of the two MDRS subscales, namely, a Multi‐Indicator Multi‐Cause model (MIMIC; Chang et al., 2020). The model tested the correlations between the two new subscales and four existing and previously validated instruments (postnatal depression [EPDS], childbirth‐related PTSD [PCL‐5], obsessive‐compulsive disorder [OCI‐R], and general symptoms of dissociation [TSI]). In this model, we considered the MDRS items as ordinal, while the other four instruments were calculated indicators. Overall, all four indicators were positively and significantly associated with the two latent MDRS factors, with associations ranging from 0.37 to 64 (*p* < 0.001). Moreover, the correlation between the MDRS subscales was like that in the subsample (Pearson's: *r* = 0.491, *p* < 0.001; Spearman's: *r* = 0.491, *p* < 0.001), where the Spearman correlation was conducted on a categorical (discrete) form of the indicators. In all further analyses, the continuous form of the MDRS subscales was used.

**Table 5:** Standardized correlations between MDRS subscales and psychopathology scales (*N* = 455)

	Postnatal depression	Childbirth‐related PTSD	OCD	General dissociative symptoms
Dissociative experiences	0.55*** (0.04)	0.45*** (0.04)	0.37*** (0.05)	0.64*** (0.03)
Intrusive thoughts	0.56*** (0.03)	0.48*** (0.03)	0.50*** (0.03)	0.53*** (0.03)
Mean	1.59	0.40	0.71	0.57
Standard deviation	0.47	0.34	0.58	0.68

*Note*: *χ* ^2^ = 153.88, *df *= 43, *p* < 0.001, *χ* ^2^/*df* = 3.58, CFI = 0.966, TLI = 0.947, RMSEA = 0.075.

Abbreviations: CFI, comparative fit index; MDRS, Maternal Disintegrative Responses Scale; OCD, obsessive‐compulsive disorder; PTSD, post‐traumatic stress disorder; RMSEA, root mean square error of approximation; TLI, Tucker–Lewis Index.

*** *p* < 0.001.

### 3.6 Dissociative experiences and intrusive thoughts by parity and infant age

The subscales were further examined by testing the effects of infant age and parity on the level of dissociative experiences and intrusive thoughts. Table 6 shows the standardized effects of these variables on the two MDRS outcomes. The results supported our expectation that multiparous mothers would experience less MDRS of the two types in comparison to primiparous mothers (dissociative experiences: beta = −0.16, *p* < 0.01; intrusive thoughts: beta = −0.23, *p* < 0.001), Also as expected, mothers of older infants experienced less dissociative experiences (beta = −0.20, *p* < 0.001), but no difference was found for age of infant on the level of intrusive thoughts. Finally, the online power calculator was used to assess model power (Preacher & Coffman, 2006). The power for all 455 participants was 0.99, with the random subsamples also producing an accepted power (1‐β = 0.87).

**Table 6:** Standardized effects of primiparous versus multiparous women and mothers of younger versus older infants on MDRS subscales (*n* = 455)

Effect	Dissociative experiences	Intrusive thoughts
Multiparous versus Primiparous	−0.16** (0.05)	−0.23***(0.05)
Infant age 4–12 months versus 0–3	−0.20*** (0.05)	−0.05 (0.05)

*Note*: *χ* ^2^ = 88.86, *df *= 31, *p* < 0.001, *χ* ^2^/*df *= 2.87, CFI = 0.982, TLI = 0.975, RMSEA = 0.064.

Abbreviations: CFI, comparative fit index; MDRS, Maternal Disintegrative Responses Scale; RMSEA, root mean square error of approximation; TLI, Tucker–Lewis Index.

** *p* < 0.01

*** *p* < 0.001.

## 4 DISCUSSION

The aim of this study was to develop and validate the MDRS, an instrument assessing the wide range of mothers' disintegrative responses that may occur in the postpartum period. In addition, relying on the theoretical literature (Main & Hesse, 1990, 1995), the new scale examines both intrusive thoughts and dissociative experiences, two aspects of disintegrative responses which, to the best of our knowledge, have never previously been measured together in a single self‐report tool. The analysis indicated good psychometric properties for the MDRS, suggesting that it is empirically valid.

Although the two factors of the scale, intrusive thoughts, and dissociative experiences, were found to be strongly correlated, both EFA and CFA supported a bidimensional construct, showing that they are separate and distinct responses. Furthermore, while the means of the two factors were relatively low, the prevalence of mothers who reported occasionally experiencing at least one of the items on each subscale was considerably high for intrusive thoughts and moderately high for dissociative experiences. The prevalence of intrusive thoughts among the mothers is consistent with previous studies (Abramowitz et al., 2003; Wynter et al., 2013) and highlights the importance of a scale that can measure this disturbing phenomenon easily and effectively among the general population, rather than as a diagnostic tool. In addition, data on the prevalence of dissociative experiences show that they are not uncommon, thus revealing the pervasiveness of experiences that may evoke a wide and powerful range of emotions among mothers and which have not previously been addressed. However, since as far as we know, this study is the first to present data on dissociative experiences specifically as manifested in a mother's care of her infant, further research is needed to better understand their prevalence in other samples as well, and to identify the circumstances that may impact them.

Analysis of the convergent and discriminant validity of the MDRS factors revealed that both were associated with several variables indicative of postpartum psychopathology, specifically, postnatal depression and childbirth‐related PTSD. However, although both factors were also found to be significantly related to OCD and general dissociative symptoms, their relationships with these variables were not the same. In line with the literature on mothers (Abramowitz et al., 2003; Fairbrother et al., 2018; Huth‐Bocks et al., 2016; Solomon & George, 2011), intrusive thoughts were more strongly associated with OCD and dissociative experiences were more strongly associated with general symptoms of dissociation. These findings confirm that the two factors examine two separate phenomena, despite the strong relationship between them. Nevertheless, since this study examines the MDRS alongside general tools that reflect psychopathology, further studies are needed to examine its validity as compared with tools that specifically examine OCD in postpartum women, as well as tools that examine adaptive and normative dissociative experiences.

In addition, primiparous women reported higher intrusive thoughts and dissociative experiences than multiparous women, highlighting the vulnerability of women in the transition to motherhood (Müller et al., 2019; Nelson et al., 2014). It may be assumed that the stress and difficulties involved in adjusting to their new role may cause first‐time mothers to feel more overwhelmed and anxious, leading to more intrusive thoughts related to infant care (Murray & Finn, 2012). In contrast, experienced mothers are likely to have greater confidence in their maternal role, as well as a more integrative perception of their identity as a mother.

Furthermore, mothers of infants up to 3 months old reported more dissociative experiences than those whose babies were aged 4–12 months, although no differences were found with regard to intrusive thoughts. The first months of the baby's life may be a sensitive and intense period for mothers (Reck et al., 2009), requiring considerable effort to adapt to the new infant, sleep deprivation, and the high demands of early caregiving. These circumstances may evoke feelings of alienation, causing some mothers to distance themselves from the infant and from reality and consequently to report dissociative experiences. These experiences may become less frequent as the mother adapts and develops a more coherent and cohesive perception of the new baby and her changed situation. The absence of a difference with regard to intrusive thoughts might be explained by the fact that the high demands of childcare and the mother's investment and sense of responsibility do not lessen as the baby grows older, and may therefore continue to be a source of intrusive thoughts.

Several limitations of the study should be noted. First, the fact that it was conducted among women from the general population demonstrates the value of the MDRS in assessing broad‐spectrum maternal responses that are not necessarily psychopathological. However, at this initial stage, we do not have information on whether a high level of intrusive thoughts and dissociative experiences may indicate a disorder. To better understand this issue, further studies might compare our results with those obtained in clinical populations using existing diagnostic questionnaires. In addition, no data were collected here to indicate whether the participants belonged to at‐risk groups, such as those with a background of adversity and trauma, who may be even more vulnerable to postpartum distress, as reflected in a higher level of intrusive thoughts and dissociative experiences. Further studies might test the MDRS specifically in such groups, as well as examining additional personal and environmental variables that may contribute to its outcomes. Finally, this study examined women at one point in time. Future longitudinal studies could provide additional indications of its validity, as well as examine its predictive ability over time.

Despite these limitations, this study represents the first substantial step toward validation of the MDRS as a new instrument for mothers in the postpartum period. Importantly, the questionnaire is short and worded in a nonthreatening manner in an effort to dissolve the taboo around this issue, and to convey the message that intrusive thoughts and dissociative experiences are common and natural, and for the most part do not reflect psychopathology.

There is promising evidence to suggest that the MDRS is a reliable and valid measure, and can therefore facilitate understanding of more latent and suppressed expressions of distress and negative responses among mothers in a variety of populations in the postpartum period. Consequently, it can allow for a new approach to the empirical examination of these phenomena. Moreover, it can aid in the design of professional interventions to help women better understand their experiences and responses when they are caring for their infant and to encourage sharing, thereby contributing to their maternal confidence and well‐being.

### PEER REVIEW

The peer review history for this article is available at https://publons.com/publon/10.1002/jclp.23414

## Data Availability

The data that support the findings of this study are available from the corresponding author upon reasonable request.

## APPENDIX A MATERNAL DISINTEGRATIVE RESPONSES SCALE

### A.1

Every woman experiences the period following childbirth in her own way. Below is a list of natural thoughts and feelings that women commonly describe having during this period. Please indicate how often you have experienced each of them in the past month.

**Table:**

	Never	Rarely	Sometimes	Often	Very often
1. When I'm with the baby, I feel that what's happening isn't real, as if it's a dream or a movie.	0	1	2	3	4
2. I think about things that could happen to my baby that aren't really logical.	0	1	2	3	4
3. When I'm with the baby or caring for him/her, I feel as if I'm not really there but only watching from a distance.	0	1	2	3	4
4. I have unwanted thoughts about bad things that could happen to my baby.	0	1	2	3	4
5. When I hold the baby, I feel as if it's not really me doing it.	0	1	2	3	4
6. When I'm caring for the baby, disturbing thoughts suddenly pop into my head.	0	1	2	3	4
7. When I'm holding the baby, the uncontrollable thought that I'm going to drop him/her flits through my mind.	0	1	2	3	4
8. There are moments when I feel like a stranger to my own baby.	0	1	2	3	4
